# Space-time clustering of childhood malaria at the household level: a dynamic cohort in a Mali village

**DOI:** 10.1186/1471-2458-6-286

**Published:** 2006-11-21

**Authors:** Jean Gaudart, Belco Poudiougou, Alassane Dicko, Stéphane Ranque, Ousmane Toure, Issaka Sagara, Mouctar Diallo, Sory Diawara, Amed Ouattara, Mahamadou Diakite, Ogobara K Doumbo

**Affiliations:** 1 Medical Statistics and Informatics Research Team, LIF -UMR 6166- CNRS/Aix-Marseille University, Faculty of Medicine, 27 Bd Jean Moulin 13385 Marseille Cedex 05, France; 2 Immunology and Genetics of Parasitic Diseases, UMR 399- INSERM/Aix-Marseille University, Faculty of Medicine, Marseille, France; 3 Malaria Research and Training Centre, Department of Epidemiology of Parasitic Diseases, Faculty of Medicine, Pharmacy and Odonto-Stomatology, University of, Bamako, Mali, BP 1805 Bamako, Mali

## Abstract

**Background:**

Spatial and temporal heterogeneities in the risk of malaria have led the WHO to recommend fine-scale stratification of the epidemiological situation, making it possible to set up actions and clinical or basic researches targeting high-risk zones. Before initiating such studies it is necessary to define local patterns of malaria transmission and infection (in time and in space) in order to facilitate selection of the appropriate study population and the intervention allocation. The aim of this study was to identify, spatially and temporally, high-risk zones of malaria, at the household level (resolution of 1 to 3 m).

**Methods:**

This study took place in a Malian village with hyperendemic seasonal transmission as part of Mali-Tulane Tropical Medicine Research Center (NIAID/NIH). The study design was a dynamic cohort (22 surveys, from June 1996 to June 2001) on about 1300 children (<12 years) distributed between 173 households localized by GPS. We used the computed parasitological data to analyzed levels of *Plasmodium falciparum, P. malariae *and *P. ovale *infection and *P. falciparum *gametocyte carriage by means of time series and Kulldorff's scan statistic for space-time cluster detection.

**Results:**

The time series analysis determined that malaria parasitemia (primarily *P. falciparum*) was persistently present throughout the population with the expected seasonal variability pattern and a downward temporal trend. We identified six high-risk clusters of *P. falciparum *infection, some of which persisted despite an overall tendency towards a decrease in risk. The first high-risk cluster of *P. falciparum *infection (rate ratio = 14.161) was detected from September 1996 to October 1996, in the north of the village.

**Conclusion:**

This study showed that, although infection proportions tended to decrease, high-risk zones persisted in the village particularly near temporal backwaters. Analysis of this heterogeneity at the household scale by GIS methods lead to target preventive actions more accurately on the high-risk zones identified. This mapping of malaria risk makes it possible to orient control programs, treating the high-risk zones identified as a matter of priority, and to improve the planning of intervention trials or research studies on malaria.

## Background

Malaria is one of the leading causes of morbidity and mortality in the world. Indeed, more than 2.4 billion people are exposed to the risk of malaria [1]. The incidence of malaria worldwide is estimated at 300 to 500 million cases per year, with 90% of these cases occurring in sub-saharan Africa. Malaria kills between 1.1 and 2.7 million people per year, including almost one million children under the age of five years in Sub-Saharan Africa [1,2]. The impact of this disease, not only in terms of mortality and morbidity, but also in terms of economic and social losses, led the United Nations to make the fight against malaria one of the priorities of its Special Initiative on Africa. The persistence of malaria despite the many control programs is due partly to the very high costs of monitoring, which are particularly difficult for developing countries to bear [3,4]. In places, the relaxation of monitoring measures has even led to increases in disease levels. The methods of control recommended by the WHO [1] are based on chemical and physicochemical control of the vector (insecticide or larvicide applications, use of mosquito nets impregnated with insect repellent), environmental modification (e.g. draining of backwaters), chemical prophylaxis (essentially in pregnant women and travelers) and the early detection, containment and prevention of epidemics. In addition, major progress in research has led to the development of several candidate vaccines, which are currently in clinical trials [5-7]. However, these control methods are expensive and therefore cannot be implemented on a large scale and in a sustained fashion in the economic context of developing countries [8]. In addition, the large-scale use of anti-vectorial measures and anti-malarial prophylaxis may lead to resistance or adaptations in the vector and in the parasite. The setting up of anti-malaria actions targeting specific zones is therefore a priority. Indeed, since 1984 the WHO has recommended control measures integrated into primary healthcare, favoring local involvement [9]. Anti-malaria actions, whether involving prevention, treatment or epidemiological information, are based at local level. Taking into account the complexity of malaria, precise research studies are needed, such as research on physiopathology, immunology or genetic susceptibility to malaria or such as intervention trials to evaluate treatments or prophylactic measures (drug, vaccine, anti-vectorial devices), in order to improve our understanding of the disease and the control [10]. Particularly, sites for malaria vaccine field trials must be precisely prepared [11]. This requires a precise knowledge of the geographic zones at risk, the levels of risk, the various risk factors and the exposed populations. The highly focal nature of malaria epidemics results in marked heterogeneity, even at the scale of a village [12]. The risk of *Plasmodium falciparum *infection is highly variable over space and time [13]. An analysis of the local epidemiological situation is therefore essential and such analyses formed one of the priorities of the 18^th ^WHO Report [3], reiterated in the 20^th ^WHO Report [1]. The WHO recommends the stratification of malaria risk. This involves an analysis of local variations, making it possible to define high-risk zones on a fine geographical scale, with the aim of increasing the efficacy of anti-malaria measures [14].

The development of geographical information systems (GIS) has been an indispensable asset to this approach [12]. Together with the progress of statistical methods for spatial analysis, GIS have improved studies for the detection of clusters at high risk of diseases over space and time. However, despite the increasing number of studies reporting on temporal or spatial changes in malaria risk, few studies have analyzed this risk at a fine scale (below district level) [4,12].

Research studies on malaria disease and intervention trials, such as vaccine trials, can be improved by such a precise epidemiological modeling. Before initiating such studies it is necessary to define local patterns and predictors of malaria transmission and infection (in time and in space). This will facilitate the selection of the appropriate study population, the intervention allocation and will enhance the accuracy and the efficiency of the analysis describing the impacts of the studied interventions.

Therefore, this study aimed to identify malaria risk at household level (resolution of 1 to 3 m), and to evaluate changes in this risk over time, in a hyperendemic village in Mali. These efforts to identify high-risk zones in space and time were designed to make it possible to identify the population at risk and local risk factors, in order to plan vaccine trials.

## Methods

### Study location

The study took place in the village of Bancoumana, located in the Sahelian zone of the Upper Niger valley (district of Kati) about 60 km south west of Bamako, the capital of Mali. This village covers an area of 2.5 km^2 ^and has a population of 8000 people [15]. The principal activities are the cultivation of rice and vegetables on the banks of the River Niger. Bancoumana is a village of hyperendemic seasonally transmitted malaria [15,16]. During the rainy season (from June to October, with temperatures of 25 to 40°C), the rate of malaria transmission is high. This rate decreases slowly thereafter, reaching a minimum in the middle of the dry season (around the month of February). Three species of *Plasmodium *are present: *P.falciparum*, *P. ovale *and *P. malariae*. *P. falciparum *displays strong predominance, accounting for more than 85% of the parasites found [16].

### Population and study design

**Figure 1:**
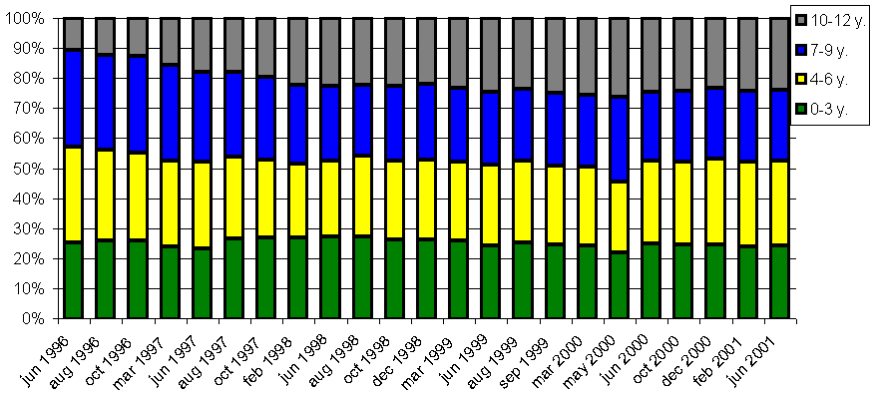
**Evolution of the age distribution of the dynamic cohort over time**. x-axis: time (date); y-axis: percentage of children for each age group.

A dynamic cohort was constituted in June 1996 and followed up until June 2001. The study included 173 of the 340 households, selected at random from each of the four geographic blocks of the village, using a stratified sampling. In each household, all the children aged 0 to 12 years were followed up, constituting the dynamic cohort (mean: 1356.68 children per survey; 95%CI [1298.98–1414.39]) with 1101 children for the first survey (June 1996) and 1491 children for the last survey (June 2001). There was therefore a mean of 9.12 children per household and per survey (95% CI [8.01–10.2]). Very few children have left the village and some are born during the study. The age distribution did not change over time and the dynamic cohort remained representative of the children population (fig. 1). The surveys (22) were carried out at the rate of about one survey every two months during the rainy season and one every three months during the dry season. The intervals between surveys were defined on the basis of the previous knowledge of the seasonal transmission [15,16].

Communal consent was first obtained. Then informed oral consent was sought from the parents or guardians of each child included, as described by Doumbo [17]. Three families refused to participate. The entire study was approved by the Institutional Committee on Ethics of the Mali Faculty of Medicine, Pharmacy, and Dentistry at the University of Bamako.

### Variables

For each survey, a blood sample was taken and parasitemia assessed. A trained team of biologists carried out microscopy to search for *P. falciparum *and its gametocytes, *P. ovale *and *P. malariae *in Giemsa-stained thick blood films. To control the quality of slide reading, a set of 10% of the blood films (randomly selected at each survey) was read by another senior biologist. In case of disagreement the senior biologist read the entire sample of blood films. Infection was defined as the presence of the parasite in the thick blood film. The medical team treated children with mild malaria: chloroquine (25 mg/kg during 3 days) was used as the first-line treatment, according to the policy of the National Malaria Control Program, at that time. The WHO 14 days *in vivo *drug test was using to assess treatment efficacy [18].

Thus, together with the intervals between surveys and with the dynamic of Good Clinical Therapeutic Responses, a second positive film at the time of a second survey would correspond to another infection (re-infection) and not to a persistent infection.

The medical team permanently stayed in the village. In any case, appropriate care was given, including hospitalization in the national hospital in Bamako if necessary.

Each child was georeferenced by household (the place where the child slept). Georeferencing was carried out using GPS (global positioning system) GeoExplorerII mapping system (1 to 3 meters accuracy) and GIS-ArcGIS8.3. This study focused on the spatial and temporal analysis of malaria infection incidence, defined as the proportion of re-infection (new positive thick blood films) per household and per survey. Therefore the first survey (June 1996) was used to control that each children was not initially infected (disease free or treated). This survey was removed from the statistical modeling.

The major risk factors in this village were: age, access to treatment, thatched roofs [19], seasonality, and presence of *Anopheles *breeding sites (varying with time even during the same season). First, to take the age factor into account in the study design, the inclusion was limited to children. Second, because physicians were present during the study, the access to the treatment was similar for each child. Third, some of the dwellings were roofed with metal sheets or (exceptionally) cement, others were thatched (47%). As the presence of malaria vectors depends on the presence of thatched roof, the spatio-temporal analyses have taken into account this covariate [15,19]. Fourth, the seasonality was taken into account by the statistical models. Fifth, our spatio-temporal analysis provided detections of high-risk zones such as *Anopheles *breeding sites.

### Statistical analysis

We began by carrying out a global temporal analysis, using classical ARIMA time series analysis models [20,21] after logarithmic transformation of the incidences. These models have been used to model time series, by breakdown into tendency, cyclic, seasonal and accidental components. The analysis was carried out with SPSS 11.5 (SPSS Inc. Chicago, IL). The models were chosen according to the criteria of Akaïke (AIC) and Schwartz (BIC).

We then looked for space-time clusters, using Kulldorff's scan statistic implemented in the Satscan program [22,23]. Widely applied [24-28] Kulldorff's Satscan program presents the advantage of using a simple statistic for identifying spatial or space-time clusters, based on geographic coordinates, that can be adjusted according to covariates. This method have been used to scan the map and time intervals, using a cylindrical window with a circular geographic base centered on each location (the radius varying from zero to an upper predetermined limit) and with height corresponding to time. The window was then moved in space and time, so that for each possible location and size, it also scanned each possible period and thus constituted spatio-temporal clusters of possible cases. High-risk cluster detection was performed by comparing the observed number of cases within the window with the expected number, using a space-time permutation model, adjusted for temporal trends and variations [29]. The number of expected cases was estimated according to the assumption of a constant risk (Poisson) distribution [22]. The rate ratio (RR) was defined as the ratio of observed to expected cases. Spatio-temporal clusters were therefore identified if, a significant excess of cases had been observed in a geographic zone during a period. The test of significance was based on a Poisson generalized likelihood ratio test, using Monte-Carlo inference. The null hypothesis of no cluster was rejected when the simulated p-value was less than or equal to 0.1. For Monte-Carlo inference, 999 replications were performed. The unit of space was defined by the coordinates of the households and the unit of time was one month. The maximum size of the time frame was 50% of the study period. We calculated 95% confidence intervals of percentages, using Wilson's method [30].

## Results

### Time series

During the five years of the study, 22 surveys were carried out, resulting in the analysis of 31200 thick blood films. We identified 13861 cases of *P. falciparum *infection over the entire study period (including 1594 cases of blood films positive for gametocytes), 612 cases of *P. malariae *infection and 185 cases of *P. ovale *infection.

Chloroquine was efficacious against falciparum malaria during the study period. The dynamic of rate of Good Clinical Therapeutic Responses was 86.7% in 1996, 88.3% in 1997, 97.2 in 1998, 97.1% in 1999, 94.4% in 2000 and 92.5% in 2001.

**Figure 2:**
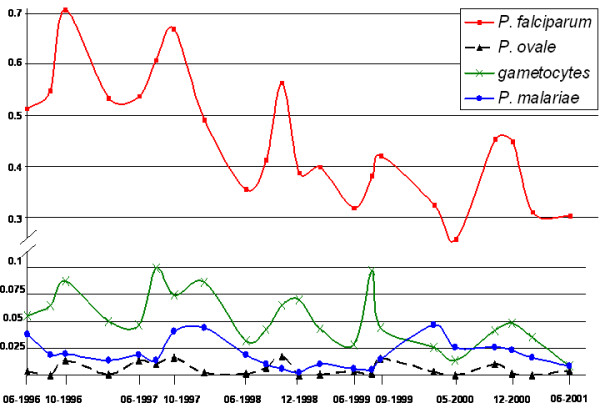
**Changes in the incidence of the three *Plasmodium *species and *P. falciparum *gametocyte in children**. x-axis: time (date); y-axis: percentage of newly infected children.

**Figure 3:**
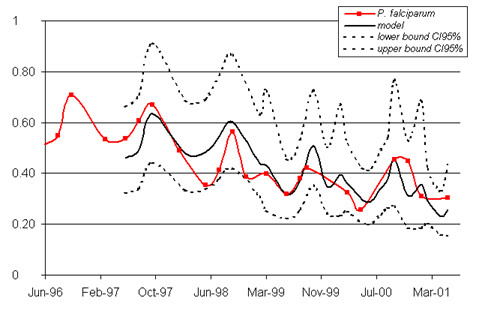
**Modeling of changes in the incidence of *P. falciparum *infection**. The model (including seasonality and a constant decrease in infection incidence from year to year) is presented in bold. The bounds of the 95% confidence interval are indicated as dotted lines. The observed data are shown as a solid line with squares to mark the observation points. x-axis: time (date); y-axis: percentage of newly infected children.

**Figure 4:**
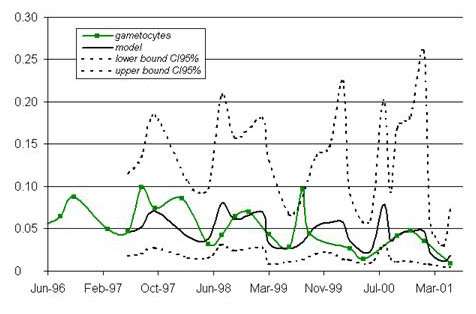
**Modeling of changes in *P. falciparum *gametocyte carriage**. The models (including seasonality and a constant decrease in infection incidence from year to year) are presented in bold. The bounds of the 95% confidence interval are indicated as dotted lines. The observed data are shown as a solid line with squares to mark the observation points. x-axis: time (date); y-axis: percentage of newly infected children.

*P. falciparum *infection incidence displayed a clear seasonal pattern on modeling (fig. 2). The constant decrease in infection from year to year was significant (p = 0.01), but remained weak (-0.107 after logarithmic transformation, standard deviation (SD) = 0.037) (fig. 3). A similar model was obtained for *P. falciparum *gametocyte carriage, with a seasonal pattern and slight decrease (constant = -0.205, SD = 0.096, p = 0.05) (fig. 4).

**Figure 5:**
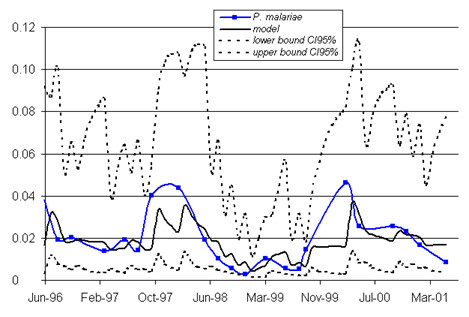
**Modeling of changes in the incidence of *P. malariae *infection**. The models (including seasonality and a constant decrease in infection incidence from year to year) are presented in bold. The bounds of the 95% confidence interval are indicated as dotted lines. The observed data are shown as a solid line with squares to mark the observation points. x-axis: time (date); y-axis: percentage of newly infected children.

An analysis of changes in the *P. malariae *incidence demonstrated a significant, first-order, autoregressive component (AR1) displaying a constant decrease (AR1 = 0.782, SD = 0.079, p < 0.0001; constant = -4.085, SD = 0.272, p < 0.0001) (fig. 5), with no significant seasonal component. The incident cases of infection with *P. ovale *were too few (less than 2.5%) for a pattern of change to be identified.

### Space-time analysis

**Figure 6:**
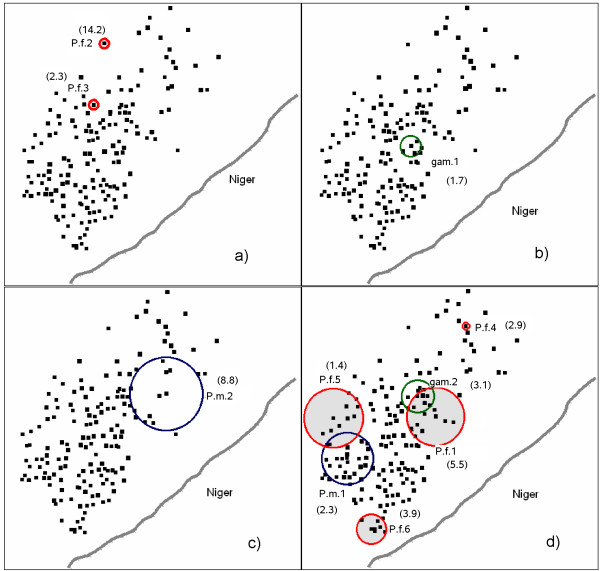
**Spatial and temporal locations of infection clusters: (a) **October 1996, **(b) **October 1997, **(c) **December 1998, **(d) **May 2000. dots represent the households. Rate Ratio are presented in brackets near each cluster. P.f.: cluster of *P. falciparum *infections (in red). gam: cluster of *P. falciparum *gametocyte carriages (in green). P.m.: cluster of *P. malariae *infections (in blue). The four time frames were selected such that all the clusters were represented. For each of the 4 time frames, the x- and y-axis represent the GPS coordinates.

The search for space-time clusters of *P. falciparum *infection demonstrated heterogeneity in both time and space. Indeed, we identified 6 significant clusters, at an α risk of 10% (Table 1). Four of the clusters occurred around 2000 and two occurred around 1996. Cluster 2, which was associated with the highest risk of malaria (ratio of observed to expected cases RR = 14.161), extended over 2 month (September and October 1996) and concerned a single household in the north of the village. Cluster 4 extended over a long period, from October 1999 to February 2001, with a high rate ratio (ratio of observed to expected cases RR = 2.92). This cluster concerned a single household in the northeast of the village (fig. 6). Cluster 5 was the largest, with a radius of 0.2 km (11 households) and was located in the west of the village. It presented a moderately high rate ratio (RR = 1.4). This cluster extended from September 1999 to June 2000. Clusters were identified in both the rainy and dry seasons, with some extending over several seasons. Furthermore, clusters did not necessarily correspond to peaks or troughs in the time series.

For *P. falciparum *gametocyte carriages (table 2), the analysis identified two time clusters, located close together in space (about 200 m apart on the ground). The first began at the end of cluster 2 for *P. falciparum *infections (i.e. in November 1996), about 300 m away (fig. 6a and 6b), with a moderately high rate ratio (RR = 1.65). The second cluster began one month before cluster 4 for *P. falciparum *infections (i.e. September 1999), 600 m away, with a high rate ratio (RR = 3.08). It extended until May 2000 and was therefore contemporary to clusters 1, 4, 5 and 6 for *P. falciparum *infection.

*P. malariae *presented two space-time clusters of infection significant at an α risk of 10% (table 3). The first, with a rate ratio of 2.27, was located in the southwest of the village and extended from October 1999 to June 2000. It therefore occurred at a similar time and in a similar place to most of the *P. falciparum *infection and gametocyte carriage clusters. The second cluster of *P.malariae *infections had a very high rate ratio (8.82). It was isolated in time, extending from September 1998 to June 1999. This cluster was located in the east of the village, in a zone in which other clusters were found at different times (clusters 1 and 2 for gametocyte carriage and cluster 1 for *P. falciparum *infection).

Finally, an analysis of *P. ovale *infection identified no significant space-time clusters (data not shown).

## Discussion

By identifying high-risk zones of malaria, this study made it possibly to stratify local risk temporally and spatially, as recommended by the WHO [1,2]. Although the entire region is classified as a high-risk zone for malaria (MARA prevalence estimation = 62.27%; 95%CI [56.37%;68.18%]) [31], the identification of clusters demonstrates the high variability of malaria risk over space and time in this village. The use of a GIS made it possible to analyze these variations precisely, at the level of households (resolution of 1 to 3 m), improving our knowledge of the disease in this village, thereby facilitating its control and its understanding, above all to plan anti-malaria intervention trials.

The time series of *P. falciparum *incidences are consistent with the well-known seasonality of infection (strongly linked to the rainy season), with marked regularity. Indeed, incidence peaked in October or September. We noted a persistence of high incidence into the start of 2000, due to the rains occurring in January 2000. Overall, the re-infection with *P. falciparum *peaked at a maximum of almost 70% (95%CI [68.1%-73.3%]) of the children studied (October 1996).

The incidence of carriage of *P. falciparum *gametocytes changed in a much less regular manner over time, notably, peaks in February and December in 1998. The peak in August 1999 was very large, exceeding the upper bound of the 95%CI. No such abrupt change was seen in changes in the *P. falciparum *incidence. There is presumably a link between this peak in the gametocyte carriage prevalence and the lengthening of the epidemic period in 1999.

A tendency towards decreasing *P. falciparum *incidence has been reported in other studies at the same site [15,16]. This tendency is unlikely to be due to natural changes in the frequency of *P. falciparum *in the region. It is also unlikely to be due to changes in the village, particularly as the proportion of dwellings with thatched roofs remained constant (about 47%). Similarly, this tendency almost certainly does not result from changes in the number of children included over time as the number of children included was already large at the start of the study and this infection is hyperendemic in this region. The tendency of *P. falciparum *re-infection to decrease is probably linked to the presence of the medical team in a population already highly aware of the problem of malaria, and also to the treatment of all infected children. Correct usage of chloroquine as the first line drug for malaria treatment has reduced significantly the self medication in the village of Bancoumana. The proportion of malaria self medication went from 6.5% in 1997 to 3.8% in 1998, 3.7% in 1999, and 0.8% in 2000 [18]. This was able to reduce chloroquino-resistant malaria parasites at the study site of Bancoumana. By contrast, we observed much more erratic changes in incidence with *P. malariae *and *P. ovale*, not consistent with seasonal transmission.

This pattern, although similar to pattern obtained for other geographic locations [4], describe an average over the entire area studied and does not take into account the geographic heterogeneity that exists, even at the small scale of a village. At household level, we can therefore call into question the globally seasonal pattern of transmission with a tendency towards decreasing incidence.

The transmission of *P. falciparum *is linked to local factors that must be identified before initiating control programs or research studies. The change in clusters over space and time is presumably linked to spatial and temporal changes in local factors, such as temporary backwaters in particular. Note that cluster 5 for *P. falciparum *infection is located on a recent site of adobe brick production. This process involves the removal of earth for the production of bricks by local craftsmen and the resulting excavations create breeding sites for mosquitoes.

These results showed that the analysis of mean changes over time at the level of an entire village is of too low a resolution whereas the search for high-risk clusters makes it possible to find a suitable interpretation for spatial and temporal changes in *P. falciparum *infection.

We observed proximity in time and space between clusters of *P. falciparum *infections and of gametocyte carriage. These observations should alert epidemiologists in the field to the existence of this zone of particularly high risk. Similarly, the extreme proximity of the two clusters of gametocyte carriage also indicates particularly high risk of transmission to the *Anopheles *mosquitoes. The first of the two clusters of *P. malariae *infections occurred close in space and time to clusters of *P. falciparum *infection, indicating the existence of local risk factors common to these two types of infection such as breeding sites for mosquitoes. The presence of space-time clusters of *P. malariae *infection should serve as an additional alarm signal.

The detection of clusters with a high risk of infection extending over several rainy seasons suggests that if that cluster had been identified when it first appeared, it might have been possible to control the risk by means of surveys on the ground to identify risk factors and the implementation of targeted control measures for this geographic zone. Although some publications have reported epidemiological analyses at district level, few have considered finer scale analyses [32-37] and only rarely has a spatial or spatio-temporal statistical model been used [12,38-40].

In this work, we studied three endemic species of *Plasmodium*. The relationships between these species are complex [41-43], particularly as *P. falciparum *is largely predominant in Mali. However, the environmental risk factors for infections with these species are largely similar. Thus, mapping the risk of infection with *P. ovale *or *P. malariae *also provides useful information concerning the risk of infection with *P. falciparum *[44]. Furthermore, space-time analysis of these different species of *Plasmodium *could improve the understanding of their relationships. Similarly, an analysis of blood infection with *P. falciparum *gametocytes provides an indication of spatial and temporal variations in malaria transmission and improves the understanding of the transmission process.

Kulldorff 's permutation model was chosen because it has several advantages: it uses only the number of cases and their localization, with no need for population at risk data; it adjusts for confounding variables; there is no pre-selection bias since the clusters are searched with no prior hypothesis on their location, size or time period; the test statistic takes into account multiple testing and delivers a single p-value [29]. Unfortunately, it is not possible to estimate confidence intervals for the rate ratios of clusters detected by scan statistics, because of the multiple testing part of the many circles evaluated.

The environmental measures recommended by the WHO [1] provide selective and targeted means of malaria control. In particular, the specific management of an environment favoring the proliferation of vectors can significantly decrease transmission [14]. The choice of interventions and their relative importance are determined by our understanding of environmental heterogeneity [8,45-47] at a sufficiently fine scale. Furthermore, in front of the high complexity of malaria transmission and infection, study populations and study environment have to be precisely evaluated before planning research studies and intervention trials [11]. The development of GIS has made it possible to increase this so-called "micro-epidemiological" knowledge [48]. This understanding and management of the environment could be applied in large African cities. The towns of Sub-Saharan Africa are growing very rapidly. The urbanization is associated with poverty, and leads to an increase in the number of malaria cases. Indeed, the new quarters created tend to lack basic sanitation structures, have high-density, poor-quality housing and there are often no drains, all of which results in the emergence of *Anopheles *breeding sites [1,14,48-50]. This situation favors large increases in the number of malaria epidemics. The detailed mapping of malaria infections in these quarters at high-risk is therefore a matter of urgency, to guide targeted interventions and studies [48,49].

## Conclusion

We must remember that trends indicating a decrease in incidence describe an average over the entire area studied. This marginal analysis should not be allowed to mask the heterogeneous distribution of malaria. Indeed, despite the overall trend, high-risk zones may persist in villages, as shown here. Even at this scale, changes are heterogeneous and probably depend on changes in the number of mosquito breeding sites (creation and destruction, whether spontaneous or due to human activities).

Analysis at the level of households, using GIS, makes it possible to determine precisely the pattern of heterogeneity in the risk of *P. falciparum *infection and transmission. The micro-epidemiological modeling makes it possible to orient control programs, treating the high-risk zones identified as a matter of priority, and to improve the planning of intervention trials or research studies on malaria. Warranting the use of such data analysis approach, in 2006 the Malaria Vaccine Development Branch (MVDB) at the NIAID/NIH, and the Malaria Research and Training Center (MRTC) at the Department of Epidemiology of Parasitic Diseases (DEAP), University of Bamako, have set up a malaria vaccine trials site (phase I, II, III) at Bancoumana.

## Abbreviations

GIS geographic information system

GPS global positioning system

MRTC Malaria Research and Training Center

MVDB Malaria Vaccines Development Branch

DEAP Department of Epidemiology of Parasitic Diseases

TMRC Tropical Medical Research Center

NIAID National Institute of Allergy and Infectious Diseases

NIH National Institutes of Health

## Competing interests

The author(s) declare that they have no competing interests.

## Authors' contributions

JG and BP contributed equally to this work.

JG performed the statistical analysis, drafted the manuscript and participated in the interpretation of data.

BP participated in the clinical, biological data collection in the field site of Bancoumana and in the interpretation of data.

AD participated in the clinical, biological data collection in the field site of Bancoumana and participated in the GPS/GIS data collection, the data computing and the validation.

SR participated in the GPS/GIS data collection, the data computing and the validation, and in the interpretation of data.

OT participated in the clinical, biological data collection in the field site of Bancoumana and participated in the GPS/GIS data collection, the data computing and the validation.

IS participated in the clinical, biological data collection in the field site of Bancoumana and participated in the QA/QC of the Data.

MDiallo participated in the clinical, biological data collection in the field site of Bancoumana and participated in the QA/QC of the malaria slides.

SD participated in the clinical, biological data collection in the field site of Bancoumana.

AO participated in the clinical, biological data collection in the field site of Bancoumana.

MDiakite participated in the clinical, biological data collection in the field site of Bancoumana and participated in the QA/QC of the malaria slides.

OKD the PI of the Mali-Tulane TMRC led the team who conceived and design the studies. He participated in the community consent protocol, in data collection, data monitoring, QA/QC of the data, data analysis and correction of the manuscript.

All authors read and approved the final manuscript.

## Pre-publication history

The pre-publication history for this paper can be accessed here:



## Figures and Tables

**Table 1:** Space-time clusters of infection with *P. falciparum*.

	Cluster			Rate Ratio (Obs/Exp)^†^	Surv^§^	Loc^¶^	p**
					
	Coordinates*	Radius Km	Time frame				
1	X = -8.26398 Y = 12.206213	0.18	2000/04 2000/05	5.495 (26/4.73)	1	15	0.001
2	X = -8.26605 Y = 12.211784	0	1996/09 1996/10	14.161 (8/0.56)	1	1	0.001
3	X = -8.2667 Y = 12.207973	0	1996/07 1996/10	2.298 (53/23.99)	2	1	0.002
4	X = -8.2621 Y = 12.211801	0	1999/10 2001/02	2.924 (30/10.26)	5	1	0.004
5	X = -8.27033 Y = 12.206117	0.2	1999/09 2000/06	1.406 (222/158.19)	3	11	0.007
6	X = -8.26797 Y = 12.199266	0.09	2000/04 2000/05	3.891 (15/3.85)	1	7	0.08

*: GPS coordinates of the centre of the cluster

†: obs: number of cases observed in the cluster; exp: number of cases expected under the null hypothesis.

§ number of survey during the time period.

¶ number of locations

**: p-value (α = 10%).

**Table 2:** Space-time clusters of infection with *P.falciparum *gametocyte carriage.

	Cluster			Rate Ratio (Obs/Exp)^†^	Surv^§^	Loc^¶^	p**
					
	Coordinates*	Radius Km	Time frame				
1	X = -8.26548 Y = 12.205422	0.07	1996/11 1998/08	1.65 (76/46.05)	7	5	0.068
2	X = -8.2651 Y = 12.207458	0.1	1999/09 2000/05	3.08 (18/5.84)	3	11	0.095

*: GPS coordinates of the centre of the cluster

†: obs: number of cases observed in the cluster; exp: number of cases expected under the null hypothesis.

§ number of survey during the time period.

¶ number of locations

**: p-value (α = 10%)

**Table 3:** Space-time clusters of infection with *P.malariae*.

	Cluster			Rate Ratio (Obs/Exp)^†^	Surv^§^	Loc^¶^	p**
					
	Coordinates*	Radius Km	Time frame				
1	X = -8.26947 Y = 12.203629	0.17	1999/10 2000/06	2.27 (30/13.21)	3	24	0.066
2	X = -8.26205 Y = 12.207684	0.240	1998/09 1999/06	8.82 (6/0.68)	4	9	0.094

*: GPS coordinates of the centre of the cluster

†: obs: number of cases observed in the cluster; exp: number of cases expected under the null hypothesis.

§ number of survey during the time period.

¶ number of locations

**: p-value (α = 10%)

## References

[B1] World Health Organization Expert Committee on Malaria (2000). 20th Report. World Health Organ Tech Rep.

[B2] Breman JG, Alilio MS, Mills A (2004). Conquering the intolerable burden of malaria: what's new, what's needed: a summary. Am J Trop Med Hyg.

[B3] World Health Organization Expert Committee on Malaria (1986). 18th Report. World Health Organ Tech Rep.

[B4] Alilio MS, Kitua A, Njunwa K, Medina M, Rønn AM, Mhina J, Msuya F, Mahundi J, Depinay JM, Whyte S, Krasnik A, Bygbjerg IC (2004). Malaria control at the district level in Africa: the case of the Muheza district in northeastern Tanzania. Am J Trop Med Hyg.

[B5] Malkin EM, Diemert DJ, McArthur JH, McArthur JH, Perreault JR, Miles AP, Giersing BK, Mullen GE, Orcutt A, Muratova O, Awkal M, Zhou H, Wang J, Stowers A, Long CA, Mahanty S, Miller LH, Saul A, Durbin AP (2005). Phase 1 clinical trial of apical membrane antigen 1: an asexual blood-stage vaccine for *Plasmodium falciparum *malaria. Infect Immun.

[B6] Saul A, Lawrence G, Allworth A, Elliott S, Anderson K, Rzepczyk C, Martin LB, Taylor D, Eisen DP, Irving DO, Pye D, Crewther PE, Hodder AN, Murphy VJ, Anders RF (2005). A human phase 1 vaccine clinical trial of the *Plasmodium falciparum *malaria vaccine candidate apical membrane antigen 1 in Montanide ISA720 adjuvant. Vaccine.

[B7] Bojang KA, Milligan PJ, Pinder M, Vigneron L, Alloueche A, Kester KE, Ballou WR, Conway DJ, Reece WHH, Gothard P, Yamuah L, Delchambre M, Voss G, Greenwood BM, Hill A, McAdam KPWJ, Tornieporth N, Cohen JD, Doherty T (2001). Efficacy of RTS, S/AS02 malaria vaccine against *Plasmodium falciparum *infection in semi-immune adult men in The Gambia: a randomised trial. Lancet.

[B8] Hanson K (2004). Public and private roles in malaria control: the contributions of economic analysis. Am J Trop Med Hyg.

[B9] WHO (1984). Malaria control as part of primary health care. World Health Organ Tech Rep.

[B10] Rogier C, Fusai T, Pradines B, Trape JF (2005). Evaluating malaria attributable morbidity in endemic areas. Rev Epidemiol Sante Publique.

[B11] Kitua AY (1997). Field trials of malaria vaccines. Indian J Med Res.

[B12] Booman M, Durrheim DN, La Grange K, Martin C, Mabuza AM, Zitha A, Mbokazi FM, Fraser C, Sharp BL (2000). Using a geographical information system to plan a malaria control programme in South Africa. Bull World Health Organ.

[B13] Baird JK, Agyei SO, Utz GC, Koram K, Barcus MJ, Jones TR, Fryauff DJ, Binka FN, Hoffman SL, Nkrumah FN (2002). Seasonal malaria attack rates in infants and young children in Northern Ghana. Am J Trop Med Hyg.

[B14] Killeen GF, Seyoum A, Knols BGJ (2004). Rationalizing historical successes of malaria control in Africa in terms of mosquito resource availability management. Am J Trop Med Hyg.

[B15] Toure YT, Doumbo O, Toure A, Bagayoko M, Diallo M, Dolo A, Vernick KD, Keister DB, Muratova O, Kaslow DC (1998). Gametocyte infectivity by direct mosquito feeds in an area of seasonal malaria transmission: implications for Bancoumana, Mali, as a transmission-blocking vaccine site. Am J Trop Med Hyg.

[B16] Dolo A, Camara F, Poudiougo B, Touré A, Kouriba B, Bagayogo M, Sangaré D, Diallo M, Bosman A, Modiano D, Touré YT, Doumbo O (2003). Epidémiologie du paludisme dans un village de savane soudanienne du Mali (Bancoumana). Bull Soc Pathol Exot.

[B17] Doumbo OK (2005). It takes a village: medical research and ethics in Mali. Science.

[B18] Poudiougo B, Diawara S, Diakite M, Diallo M, Dicko A, Sagara I, Toure O, Dolo A, Krogstad D, Doumbo O (2002). The impact of community-based malaria case management on the anti-malaria drugs resistance in south of Mali: Bancoumana. 3rd MIM Pan-African Conference on Malaria, Arusha Tanzania.

[B19] Touré Y (2002). The *Anopheles gambiae *genome: potential contribution to malaria vector control. 3rd MIM Pan-African Conference on Malaria Arusha Tanzania.

[B20] Box GEP, Jenkins GM (1976). Time series analysis: forecasting and control.

[B21] Droesbeke JJ, Fichet B, Tassi P (1989). Séries chronologiques: théorie et pratique des modèles ARIMA.

[B22] Kulldorff M (1997). A spatial scan statistic. Com Stat Theory and Methods.

[B23] Satscan™ program v5.1, Information Management Services Inc., Silver Spring, Maryland, 2004; freeware. http://www.satscan.org.

[B24] Odoi A, Martin SW, Michel P, Holt J, Middleton D, Wilson J (2003). Geographical and temporal distribution of human giardiasis in Ontario, Canada. Int J Health Geogr.

[B25] Nkhoma ET, Hsu CE, Hunt VI, Harris AM (2004). Detecting spatiotemporal clusters of accidental poisoning mortality among Texas counties, U.S., 1980 – 2001. Int J Health Geogr.

[B26] Chaput EK, Meek JI, Heimer R (2002). Spatial analysis of human granulocytic Ehrlichiosis near Lyme, Connecticut. Emerg Infect Dis.

[B27] Mostashari F, Kulldorff M, Hartman JJ, Miller JR, Kulasekera V (2003). Dead bird clusters as an early warning system for West Nile virus activity. Emerg Infect Dis.

[B28] Guis H, Clerc S, Hoen B, Viel JF Clusters of autochthonous hepatitis A cases in a low endemicity area. EpidemiolInfect.

[B29] Kulldorff M, Heffernan R, Hartman J, Assunção R, Mostashari F (2005). A space-time permutation scan statistic for disease outbreak detection. PLoS Med.

[B30] Newcombe RG (1998). Two-sided confidence intervals for the single proportion: comparison of seven methods. Stat Med.

[B31] MARA/ARMA 2002 version3.0.0 build5, South Africa Medical Research Council.

[B32] Schellenberg JA, Newell JN, Snow RW, Mungala V, Marsh K, Smith PG, Hayes RJ (1998). An analysis of geographical distribution of severe malaria in children in Kilifi district, Kenya. Int J Epidemiol.

[B33] Kleinschmidt I, Sharp B, Mueller I, Vounatsou P (2002). Rise in malaria incidence rates in South Africa: a small-area spatial analysis of variation in time trends. Am J Epidemiol.

[B34] Domarle O, Migot-Nabias F, Pilkington H, Elissa N, Toure FS, Mayombo J, Cot M, Deloron P (2002). Family analysis of malaria infection in Dienga, Gabon. Am J Trop Med Hyg.

[B35] Quakyi IA, Leke RGF, Befidi-Mengue R, Tsafack M, Bomba-Nkolo D, Manga L, Tchinda V, Njeungue E, Kouontchou S, Fogako J, Nyonglema P, Thuita Harun L, Djokam R, Sama G, Eno A, Megnekou R, Metenou S, Ndoutse L, Same-Ekobo A, Alake G, Meli J, Ngu J, Tietche F, Lohoue J, Mvondo JL, Wansi E, Leke R, Folefack A, Bigoga J, Bomba-Nkolo C, Titanji V, Walker-Abbey A, Hickey MA, Johnson AH, Taylor DW (2000). The epidemiology of *Plasmodium falciparum *malaria in two Cameroonian villages: Simbok and Etoa. Am J Trop Med Hyg.

[B36] Rogier C, Ly AB, Tall A, Cisse B, Trape JF (1999). *Plasmodium falciparum *clinical malaria in Dielmo, a holoendemic area in Senegal: no influence of acquired immunity on initial symptomatology and severity of malaria attacks. Am J Trop Med Hyg.

[B37] Briet OJT, Gunawardena DM, Van der Hoek W, Amerasinghe FP (2003). Sri Lanka malaria maps. Malaria J.

[B38] Gemperli A, Vounatsou P, Kleinschmidt I, Bagayoko M, Lengeler C, Smith T (2004). Spatial patterns of infant mortality in Mali: the effect of malaria endemicity. Am J Epidemiol.

[B39] Sipe NG, Dale P (2003). Challenges in using geographic information systems (GIS) to understand and control malaria in Indonesia. Malaria J.

[B40] Smith T, Charlwood JD, Takken W, Tanner M, Spiegelhalter DJ (1995). Mapping the densities of malaria vectors within a single village. Acta Tropica.

[B41] Smith T, Genton G, Baea K, Gibson N, Narara A, Alpers MP (2001). Prospective risk of morbidity in relation to malaria infection in an area of high endemicity of multiple species of *Plasmodium*. Am J Trop Med Hyg.

[B42] May J, Mockenhaupt FP, Ademowo OG, Falusi AG, Olumese PE, Bienzle U, Meyer CG (1999). High rate of mixed and subpatent malarial infections in southwest Nigeria. Am J Trop Med Hyg.

[B43] Prybylski D, Khaliq A, Fox E, Sarwari AR, Strickland T (1999). Parasite density and malaria morbidity in the Pakistan Punjab. Am J Trop Med Hyg.

[B44] Von Seidlein L, Drakeley C, Greenwood B, Walraven G, Targett G (2001). Risk factors for gametocyte carriage in Gambian children. Am J Trop Med Hyg.

[B45] Kiszewski AE, Teklehaimanot A (2004). A review of the clinical and epidemiologic burdens of epidemic malaria. Am J Trop Med Hyg.

[B46] Chadee DD, Kitron U (1999). Spatial and temporal patterns of imported malaria cases and local transmission in Trinidad. Am J Trop Med Hyg.

[B47] Mbogo CM, Mwangangi JM, Nzovu J, Gu W, Yan G, Gunter JT, Swalm C, Keating J, Regens JL, Shililu JI, Githure JI, Beier JC (2003). Spatial and temporal heterogeneity of *Anopheles *mosquitoes and *Plasmodium falciparum *transmission along the Kenyan coast. Am J Trop Med Hyg.

[B48] Caldas de Castro M, Yamagata Y, Mtasiwa D, Tanner M, Utzinger J, Keiser J, Singer BH (2004). Integrated urban malaria control: a case study in Dar Es Salaam, Tanzania. Am J Trop Med Hyg.

[B49] Keiser J, Utzinger J, Caldas de Castro M, Smith TA, Tanner M, Singer BH (2004). Urbanization in Sub-Saharan Africa and implication for malaria control. Am J Trop Med Hyg.

[B50] Kazadi W, Sexton JD, Bigonsa M, W'Okanga B, Way M (2004). Malaria in primary school children and infants in Kinshasa, Democratic Republic of the Congo: surveys from the 1980s and 2000. Am J Trop Med Hyg.

